# The relationship between the baseline geriatric nutritional risk index (GNRI) and neurological function at the convalescence stage in patients with stroke: a cross-sectional study

**DOI:** 10.1186/s12877-023-03919-w

**Published:** 2023-03-27

**Authors:** Lielie Zhu, Jianning Xia, Xiangzhi Shao, Xinyu Pu, Jiajun Chen, Jiacheng Zhang, Xinming Wu, Jinyihui Zheng, Dengchong Wu, Bing Chen

**Affiliations:** Department of Rehabilitation, Zhejiang Chinese Medical University Affiliated Wenzhou Hospital of Traditional Chinese Medicine, Wenzhou, Zhejiang China

**Keywords:** Geriatric nutritional risk index (GNRI), Neurological outcomes, Stroke, Convalescence stage, Predictor

## Abstract

**Background:**

Malnutrition is a common complication after stroke and may worsen neurological outcomes for patients. There are still no uniform tools for screening nutritional status for the patients with stroke. We aimed to explore the relationship between the baseline geriatric nutritional risk index (GNRI) and neurological function at the convalescence stage for patients with stroke and assessed the predictive value of the GNRI for adverse neurological outcomes.

**Methods:**

A total of 311 patients with stroke were enrolled retrospectively. Basic information and laboratory results on admission since onset of stroke were collected. The GNRI on admission was calculated and neurological outcomes evaluated by the Barthel index at 1 month after the onset of stroke. Statistical analyses, including correlation coefficient tests, multivariate regression analyses, and receiver operating characteristic (ROC) analyses, were applied in this study.

**Results:**

Compared with the good outcome group, the poor outcome group showed a significantly lower GNRI on admission (*P* < 0.05). GNRI was associated with Barthel index (r = 0.702, *P* < 0.01). The GNRI was independently correlated with the Barthel index (Standardization β = 0.721, *P* < 0.01) and poor outcome 0.885 (95% CIs, 0.855–0.917, *P* < 0.01) after adjusting for covariates. Compared with no nutritional risk grades (Q4), the OR of GNRI to poor neurological outcome increased across increasing nutritional risk grades of GNRI (OR = 2.803, 95% CIs = 1.330–5.909 in Q3, 7.992, 95% CIs = 3.294–19.387 in Q2 and 14.011, 95% CIs = 3.972–49.426 in Q1, respectively, *P* for trend < 0.001). The area under ROC curves (AUC) of the GNRI was 0.804, which was larger than that of the NIHSS, BMI, or Albumin (*P* < 0.01), with an optimal cut-off value of 97.69, sensitivity of 69.51% and specificity of 77.27%. Combined GNRI with NIHSS gained the largest AUC among all the variables (all *P* < 0.05), with an AUC of 0.855, sensitivity of 84.75 and specificity of 72.73%.

**Conclusions:**

For patients with stroke, higher nutritional risk grades at baseline indicated worse neurological function at the convalescence stage. Compared with NIHSS, BMI, and Albumin, GNRI was a competitive indicator for the risk of poor neurological outcome. The predictive property of GNRI for adverse neurological outcomes might be more powerful when combined with NIHSS.

## Background

As a common complication after stroke, malnutrition is closely related to the concomitant factors of stroke, such as age, neurological defects, swallowing dysfunction, and a decline in daily living activities [1, 2]. It is reported that the incidence of malnutrition post stroke can reach 16%-66.7% [1, 3]. Malnutrition can exacerbate stroke, hinder functional recovery, prolong hospital stay, and even increase mortality [4]. For the patients with stroke at the convalescence stage, malnutrition may can also hamper rehabilitation and worsen neurological outcomes [1, 5].

A study revealed that nutritional improvement in stroke patients with malnutrition was associated with the resumption of activities of daily living [6]. However, malnutrition for patients with stroke remains incompletely recognized, which leads to an undertreated problem [4]. One simple strategy for quickly identifying patients with nutritional problems who may benefit from nutritional intervention is to use a validated nutrition screening tool (NST). Current guidelines recommend that all stroke patients should be screened for risk of malnutrition at admission [7]. Patients identified as at risk of malnutrition should be subjected to further assessment and subsequently receive an appropriate nutritional intervention [8]. It is believed that early screening or identification of malnutrition of patients with stroke and predicting the neurological outcome in stroke rehabilitation patients could facilitate appropriate nutritional intervention, which is important to regain functional capacity and activities of daily living, as well as to improve the quality of life [5].

Nevertheless, there are still few NSTs designed and validated specifically for stroke patients [2, 5]. For most of common NSTs, their prediction forpoor clinical outcome was also seldom performed based on the neurological function of stroke [9]. In recent years, the Geriatric Nutritional Risk Index (GNRI) [10], which is based on serum albumin and actual weight and ideal weight ratio, has been used as a simple and effective NST in a variety of clinical environments, such as evaluating nutritional status [2] and predicting outcome events for many illnesses, such as tumor, trauma, hemodialysis and heart failure [11–14]. However, the relationship between GNRI and adverse neurological outcomes in patients with stroke is unclear, and it is still unknown whether the GNRI plays a predictive role for adverse neurological outcomes. In this study, we explored the relationship between the GNRI and neurological function of the convalescence stage in patients with stroke and assessed the predictive value of the GNRI.

## Methods

### Study design

This was a single-centre study to explore the relationship between the GNRI and neurological functionin patients with stroke at the convalescence stage. A medical record review of the patients admitted to Zhejiang Chinese Medical University Affiliated Wenzhou Hospital of Traditional Chinese Medicine from January 2022 to September 2022 was performed. This study was approved by the Ethics Committee of Zhejiang Chinese Medical University Affiliated Wenzhou Hospital of Traditional Chinese Medicine (No.WZY2021-KT-072).

### Participants

Inclusion criteria: (1) age between 50 and 85 years; (2) met the diagnostic criteria for ischaemic stroke, listed in the Diagnostic criteria of cerebrovascular disease in China (version 2019) [15]; (3) patients admitted to the hospitals within one week after the onset of stroke confirmed by magnetic resonance imaging or computerized tomography scans; (4) the patients’ baseline data within 1 week since onset of stroke were intact; and (5) information about neurological function evaluation at 1 month after onset of stroke was available. Exclusion criteria: (1) patients who died within 1 month after the onset of stroke; and (2) patients whose medical information was missing or incomplete.

According to the above criteria,we enrolled a total of 311 patients with stroke at the convalescence stage (225 males and 86 females subjects) aged 50.5–85.5 years with complete data. All participants received the necessary supportive care for ischaemic stroke patients, including antiplatelet, lipid-lowering, blood pressure-controlling, and blood glucose-regulating medications, and at the same time, specific treatments for comorbidity were implemented according to the patient’s condition. Meanwhile, all of the participants underwent a routine rehabilitation program, which included physiotherapy, occupational therapy, physical agent therapy, and speech or swallowing therapy, depending on the patient's condition. All patients were divided into the poor outcome group (Barthel index < 60, n = 223) and the good outcome group (Barthel index ≥ 60, *n* = 88) according to the Barthel index evaluated at 1 month after the onset of stroke.

### Data collection

A retrospective review was conducted on the patients’ clinical records at baseline (within 1 weeks since the onset of stroke), including the characteristics of demographic, clinical and medical history variables, such as age, sex, height, body weight, smoking history, drinking history, comorbidity, the infarct type, thrombolytic therapy or not, CRP (C-reactive protein), serum Albumin and National Institute of Health Stroke Scale (NIHSS) scores. BMI was calculated by dividing weight (kg) by the square of height (m^2^). According to the Oxfordshire Community Stroke Study (OSCP) [16], infarction was divided into three types in this study: total anterior circulation infarct (TACI), partial anterior circulation infarction (PACI), and posterior circulation infarction (POCI). The NIHSS is a 15-item impairment scale used to measure and assess stroke severity, recommended by National Stroke Foundation guidelines. The NIHSS includes the following domains: level of consciousness, eye movements, integrity of visual fields, facial movements, arm and leg muscle strength, sensation, coordination, language, speech and neglect. Each impairment is scored on an ordinal scale ranging from 0 to 2, 0 to 3, or 0 to 4. Item scores are summed toa total score ranging from 0 to 42 (the higher the score, the moresevere the stroke) [17].

### GNRI calculation

The GNRI was calculated using the following formula [18]: $\mathrm{GNRI}=\lbrack1.489\times\mathrm{albumin}\;(\mathrm g/\mathrm l)\rbrack+41.7\times\lbrack\mathrm{body}\;\mathrm{weight}\;(\mathrm{kg})/\mathrm{ideal}\;\mathrm{body}\;\mathrm{weight}\;(\mathrm{kg})\rbrack$; the ideal weightwas calculated using the following formula: $\mathrm{Ideal}\;\mathrm{body}\;\mathrm{weight}\;(\mathrm{men})=\mathrm{height}\;(\mathrm{cm})\times0.75-62.5$; and $\mathrm{Ideal}\;\mathrm{body}\;\mathrm{weight}\;(\mathrm{women})=\mathrm{height}\;(\mathrm{cm})\times0.60-40$.

The radio of bodyweight to ideal body weight was set as “1” when the body weight exceeded the ideal body weight.

Based on the above values of GNRI, four grades of risk related to nutrition were defined and used as subgroups of GNRI for further analysis [19]: high nutritional risk (Q1): GNRI < 82; medium nutritional risk (Q2): 82 ≤ GNRI < 92; low nutritional risk (Q3): 92 ≤ GNRI ≤ 98; no nutritional risk (Q4): GNRI > 98.

### Outcome measurements

Barthel index [20]: The index is specifically divided into 10 items: eating, dressing, toilet use, stool control, urine control, going up and down stairs, bed and chair transfer, walking on the flat ground, bathing and modification. The total score of the Barthel index scale is 0 to 100. A higher score indicates better daily self-care ability. Generally, a total score of 40 indicates severe dependence, 41 to 60 indicates moderate dependence, 61 to 99 indicates mild dependence, and 100 indicates that the patients can fully care for themselves and are not dependent. According to a previous study [21], the cut-off of the Barthel index was set at 60 because patients with a Barthel index < 60 were indicated to have functional dependency. A Barthel index of < 60 was considered an indicator of poor outcome, and a Barthel index ≥ 60 was considered a good outcome, which was used as the basis for grouping in this study.

According to the regular clinical evaluation procedure, the Barthel index was evaluated at 1 months after onset of stroke which was recorded in the institutional database.

### Statistical analysis

Statistical analysis was performed using the IBM SPSS software version 23. For continuous variables, normally distributed variables were described as means ± SDs while obviously skewed variables are expressed as the median (interquartile range, IQR). The proportion or prevalence was used to describe categorical variables. The univariate analysis included *t*-test and Pearson’s χ^2^-test was used to compare the mean and proportion. Spearman partial coefficient analysis was applied to assess the correlations between GNRI and other clinical characteristics or biomarkers. Multivariate logistic regression analysis was used to analyse the independent correlation between variables and neurological outcomes. The significant variables in univariate analysis and covariates considered clinically influential or potential risk factors according to the literature published previously were then analysed by multivariate stepwise logistic regression (backwards stepwise) to identify significant variables affecting neurological outcomes. The odds ratio (OR) and 95% confidence intervals (CIs) were calculated. To evaluate the effect of biomarkers in predicting neurological outcomes, receiver operating characteristic (ROC) curves were plotted with the area under the curve (AUC) and the best cut-off values were calculated. The sensitivity and specificity were used to show the predictive value of the GNRI. Statistically significant differences were defined as a two-tailed *P* < 0.05.

## Results

### Basic characteristics

The 311 patients included in the analysis had a mean GNRI of 95.42 ± 11.97. According to the nutritional risk grades related to GNRI at baseline, there were 51 patients (16.4%) at high nutritional risk (Q1), 75 patients (24.1%) at medium nutritional risk (Q2), 54 patients (17.4%) at low nutritional risk (Q3), and 131 patients (42.1%) at no nutritional risk (Q4). The basic characteristics of the study population are presented in Table 1. Compared with the good outcome group, the poor outcome group showed a significantly higher proportion of drinking, a higher level of NIHSS scores, and lower BMI, GNRI, and Barthel index (*P* < 0.05). No significant difference in age, the proportion of sexes, smoking, the distribution of comorbidities, the infarct types, the proportion of thrombolytic therapy, or CRP level was found between the two groups (all *P* > 0.05).

**Table 1:** Basic characteristics of the subjects

Characteristic	The poor outcome group (Barthel index<60) (*n* = 223)	The good outcome group (Barthel index ≥ 60) (*n* = 88)	t/χ^2^/z	*P*
Median age (years)	67.7 ± 9.3	67.0 ± 9.1	0.680	0.497
Sex (male/female)	158/65	67/21	0.881	0.348
Smoking (Yes),[n(%)]	103(46.2)	36(40.9)	0.711	0.399
Drinking (Yes),[n(%)]	92(41.3)	20(22.7)	9.400	0.002
BMI (kg/m^2^)	22.81 ± 4.17	25.84 ± 4.77	-5.536	<0.001
Comorbidity,n(%)				
Hypertension	153(68.6)	61(69.3)	0.015	0.903
Diabetes	192(86.1)	72(81.8)	0.901	0.342
Hyperlipidaemia	158(70.9)	56(63.6)	1.531	0.216
Heart failure	12(5.4)	4(4.5)	-^a^	1.000
COPD	30(13.5)	11(12.5)	0.050	0.823
Pneumonia	29(13.0)	8(9.1)	0.922	0.337
Chronic kidney disease	21(9.4)	5(5.7)	1.149	0.284
Other diseases	38(17.0)	11(12.5)	0.980	0.322
Infarct type,[n(%)]				
TACI	46(20.6)	17(19.3)	2.127	0.345
PACI	100(44.8)	33(37.5)		
POCI	77(34.5)	38(43.2)		
Thrombolytic therapy (Yes),[n(%)]	70(31.4)	36(40.9)	2.545	0.111
NIHSS scores	14.24 ± 2.66	11.90 ± 2.39	7.195	<0.001
CRP (mg/L)	17.11(12.48,27.64)	15.10(11.54,27.60)	-1.615	0.106
Albumin(g/L)	35.17 ± 7.27	43.06 ± 7.50	-8.540	<0.001
GNRI	91.68 ± 10.18	104.87 ± 10.97	-10.065	<0.001
GNRI group,[n(%)]				
Q1(GNRI<82)	48(21.5)	3(3.4)	53.360	<0.001
Q2(82 ≤ GNRI<92)	68(30.5)	7(8.0)		
Q3(92 ≤ GNRI ≤ 98)	40(17.9)	14(15.9)		
Q4(98<GNRI)	67(30.0)	64(72.7)		
Barthel index	35.40 ± 15.00	78.01 ± 12.83	-23.464	<0.001

Other diseases included gastrointestinal haemorrhage, other infections, and pressure sores

*Abbreviations*: *BMI* body mass index, *TACI* total anterior circulation infarct, *PACI* partial anterior circulation infarction, *POCI* posterior circulation infarction, *NIHSS* National Institute of Health Stroke Scale, *GNRI* geriatric nutritional risk index

^a^ Fisher’s exact test

### Case distribution characteristics of poor and good outcome in different GNRI subgroups

**Fig. 1:**
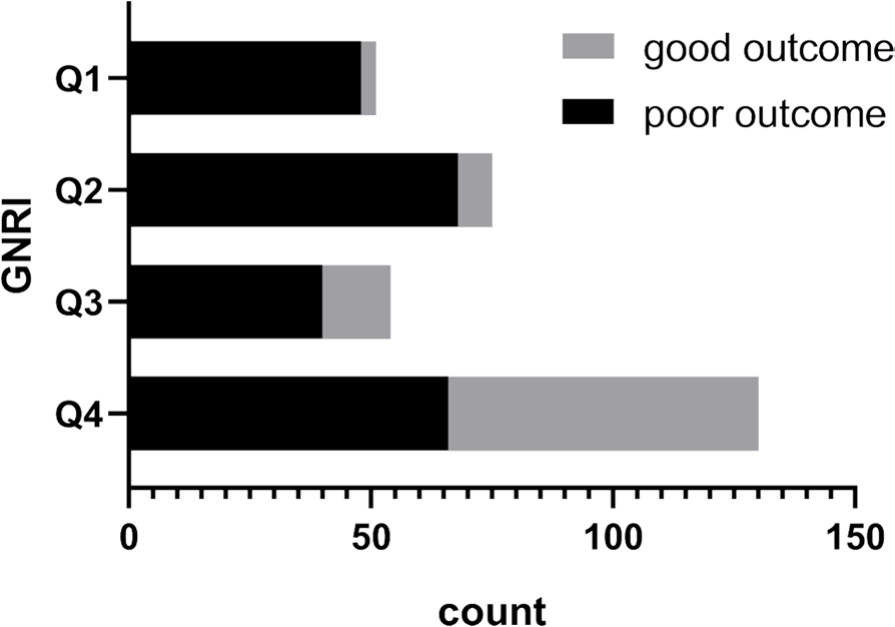
Case distribution with poor and good outcome categorized by GNRI subgroups. Abbreviations: GNRI: geriatric nutritional risk index

The constituent ratio of cases in different subgroups of GNRI between the poor outcome group and the good outcome group are presented in Table 1. In the good outcome group, more cases were distributed in the lower nutritional risk grades of GNRI (constituent ratios were 3.4%, 8.0%, 15.9%, and 72.7% in Q1, Q2, Q3, and Q4, respectively, *P* < 0.05). Additionally, it was found that most cases in the Q1 subgroup had poor outcomes (94.1%), and with elevated GNRI grades, the proportion of cases with poor outcomes decreased 90.7%, 74.1% and 51.1% in Q2, Q3, and Q4, respectively (z = 49.268, *P* < 0.001, according to linear by linear association). Meanwhile, the proportion of cases with good outcomes increased with elevated GNRI grades (5.9%, 9.3%, 25.9%, and 48.9%, respectively, *P* < 0.001), which are presented in Table 2. Figure 1 intuitively shows the case distribution of poor and good outcomes stratified by GNRI subgroups.

**Table 2:** Comparison of the case distribution between poor and good outcomes in different subgroups of GNRI

	GNRI				n	χ2	z	*P*
	Q1(GNRI<82)	Q2(82 ≤ GNRI<92)	Q3(92 ≤ GNRI ≤ 98)	Q4(98<GNRI)				
Poor outcome cases (Barthel index<60)	48(94.1%)	68(90.7%)	40(74.1%)	66(51.1%)	223	53.390	49.268▲	<0.001
Good outcome cases (Barthel index ≥ 60)	3(5.9%)	7(9.3%)	14(25.9%)	64(48.9%)	88			
n	51	75	54	131	311			

▲*P* < 0.05, according to linear by linear association

*Abbreviations*: *GNRI* geriatric nutritional risk index

### Spearman’s partial correlations between GNRI, Albumin, BMI, NIHSS and Barthel index

**Fig. 2:**
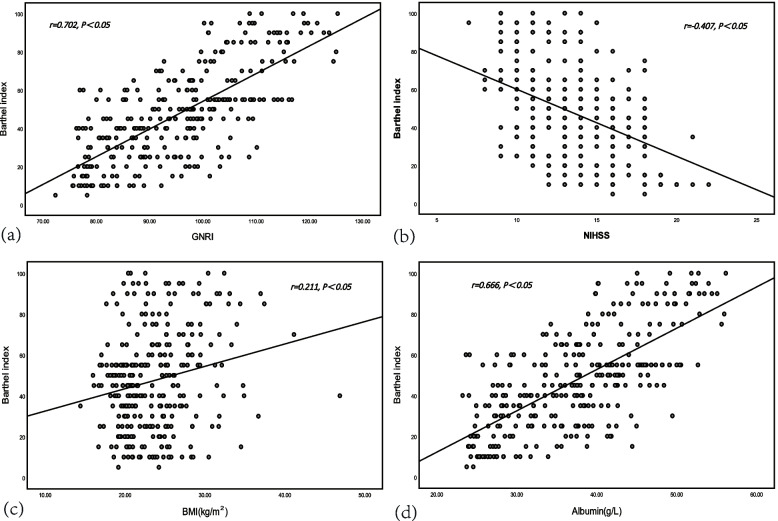
Scatter diagrams showing relationship between GNRI(a),NIHSS(b),BMI(c), Albumin(d) and Barthel index. Abbreviations: GNRI: geriatric nutritional risk index; NIHSS: National Institute of Health Stroke Scale; BMI: body mass index

After adjusting for age, sex, drinking, smoking, the infarct type, and comorbidity, Spearman’s partial correlation analysis showed that GNRI, BMI, and Albumin were all positively and significantly associated with the Barthel index (*r* = 0.702, 0.211, and 0.666, respectively, all *P* < 0.01), and NIHSS was negatively associated with the Barthel index (*r* = -0.407, *P* < 0.01). In addition, GNRI grades also showed a positive relationship similar to that of GNRI with the Barthel index (*r* = 0.611, *P* < 0.01), which verified a robust result. The details of the Spearman’s correlation analysis are presented in Table 3. The relationships between GNRI, Albumin, BMI, NIHSS and Barthel scores are presented as scatter diagrams in Fig. 2.

**Table 3:** Spearman’s correlations of between GNRI, BMI, NIHSS, Ablumin, and Barthel index

Variable	Barthel index	
	*r* ^a^	*P*
**GNRI**	0.702	<0.001
**GNRI grades**	0.611	<0.001
**NIHSS**	-0.407	<0.001
**BMI**	0.211	<0.001
**Albumin**	0.666	<0.001

*Abbreviations*: *GNRI* geriatric nutritional risk index, *NIHSS* National Institute of Health Stroke Scale, *BMI* body mass index

^a^adjusted for age, sex, drinking, smoking, the infarct type, comorbidity, thrombolytic therapy, and CRP

### Line regression analyses for the Barthel index

Table 4 shows the multivariate linear regression analysis for the Barthel index. It was shown that GNRI, NIHSS, BMI, and Albumin all correlated with the Barthel index according to the univariate analysis (all *P* < 0.01). After adjusting for age, sex, drinking, smoking, the infarct type, and comorbidity, the correlations still existed. It was found that GNRI, NIHSS, BMI, and Albumin were all independently correlated with the Barthel index (Standardization β = 0.721, -0.406, 0.205, and 0.673, respectively, all *P* < 0.01).

**Table 4:** Line regression analysis of GNRI, NIHSS, BMI, and Albumin to the Barthel index

Variables	Model1			Model2		
	β	Standardization β	*P*	β	Standardization β	*P*
**GNRI**	1.447	0.721	<0.001	1.447	0.721	<0.001
**NIHSS**	-3.518	-0.408	<0.001	-3.496	-0.406	<0.001
**BMI**	1.095	0.207	<0.001	1.081	0.205	<0.001
**Albumin**	2.027	0.687	<0.001	1.985	0.673	<0.001

Model1:crude model; Model2: adjusted for age, sex, drinking, smoking, the infarct type, comorbidity, thrombolytic therapy, and CRP

*Abbreviations*: *GNRI* geriatric nutritional risk index, *NIHSS* National Institute of Health Stroke Scale, *BMI* body mass index

### Logistic regression analyses for poor outcome

The GNRI was analysed in the logistic regression model as a continuous and categorical variable,separately. Logistic regression analyses to examine the relationship between GNRI, NIHSS, BMI, Albumin and poor outcome are listed in Table 5. In model 1 (crude model), the odds ratio(OR) of GNRI (continuous variable) for poor outcome was 0.891 (95% CIs, 0.865–0.918, *P* < 0.01), and in multivariate model 3, which adjusted for age, sex, drinking, smoking, the infarct type, comorbidity, thrombolytic therapy, CRP, BMI, and NIHSS, the OR of GNRI (continuous variable) for outcome was 0.885 (95% CIs, 0.855–0.917) (*P* < 0.01). NIHSS, BMI, and Albumin exhibited independent associations with poor outcome (OR = 1.447, 0.856, and 0.847, respectively, *P* < 0.01) after adjusting for covariates, which were similar to GNRI.

**Table 5:** Logistic regression analysis of GNRI, NIHSS, BMI, and Albumin to poor outcome

Variables	Model1			Model2			Model3		
	β	Odds ratios(95%CI)	*P*	β	Odds ratios(95%CI)	*P*	β	Odds ratios(95%CI)	*P*
**GNRI**	-0.115	0.891(0.865–0.918)	<0.001	-0.118	0.889(0.862–0.916)	<0.001	-0.122	0.885(0.855–0.917)^a^	<0.001
**NIHSS**	0.369	1.447(1.284–1.631)	<0.001	0.376	1.456(1.291–1.644)	<0.001	0.370	1.447(1.248–1.678)^b^	<0.001
**BMI**	-0.147	0.863(0.814–0.915)	<0.001	-0.146	0.865(0.814–0.919)	<0.001	-0.156	0.856(0.797–0.919)^c^	<0.001
**Albumin**	-0.140	0.869(0.836–0.904)	<0.001	-0.140	0.869(0.834–0.906)	<0.001	-0.166	0.847(0.806–0.891)^a^	<0.001

Model1:crude model; Model2: adjusted for age, sex, drinking, smoking, the infarct type, comorbidity, thrombolytic therapy, and CRP

Model3: ^a^adjusted for Model2 + NIHSS + BMI; ^b^adjusted for Model2 + GNRI + BMI; ^c^adjusted for Model2 + GNRI + NIHSS

The relationship between the GNRI grade and poor outcome was further analysed and is presented in Table 6. After adjusting for age, sex, drinking, smoking, the infarct type, comorbidity, thrombolytic therapy, and CRP, the ORs of GNRI to poor outcome across increasing nutritional risk grades were 1.00 in Q4, 2.803 (95% CIs, 1.330–5.909) in Q3, 7.992 (95% CIs, 3.294–19.387) in Q2 and 14.011 (95% CIs, 3.972–49.426) in Q1 (*P* for trend < 0.001). The details are presented in Table 6.

**Table 6:** The associations between GNRI subgroups and poor outcome

GNRI subgroups	n(%)	Model1		Model2	
		Odds ratios(95%CI)	*P*	Odds ratios(95%CI)	*P*
Q1	51(16.4)	15.284(4.532–51.545)	<0.001	14.011(3.972–49.426)	<0.001
Q2	75(24.1)	9.279(3.966–21.712)	<0.001	7.992(3.294–19.387)	<0.001
Q3	54(17.4)	2.729(1.357–5.487)	0.005	2.803(1.330–5.909)	0.007
Q4	131(42.1)	1.0	-	1.0	-
*P for trend*		<0.001		<0.001	

Model1:cruded model; Model2: adjusted for age, sex, drinking, smoking, the infarct type, comorbidity, thrombolytic therapy, and CRP

*Abbreviations*: *GNRI* geriatric nutritional risk index

### Predictive property of the GNRI for poor outcome

**Fig. 3:**
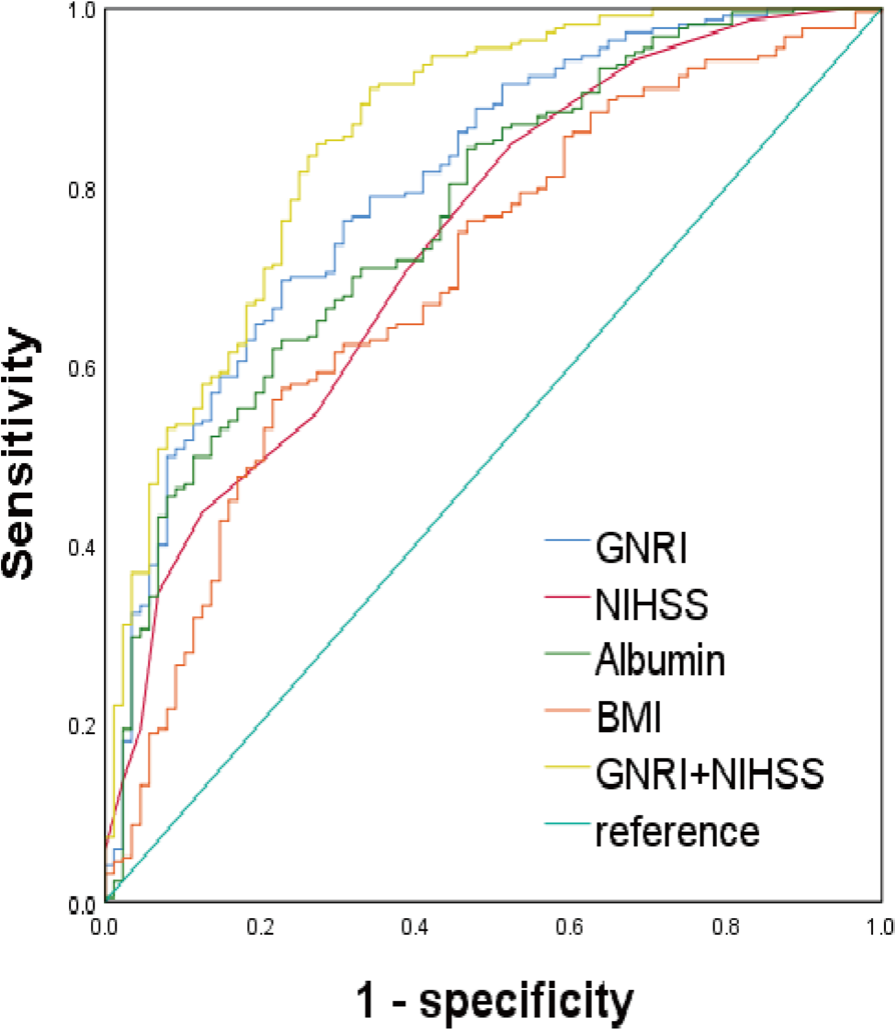
ROC analysis of GNRI, Albumin, NIHSS, BMI, and GNRI + NIHSS for poor outcome. Abbreviations: GNRI: geriatric nutritional risk index; NIHSS: National Institute of Health Stroke Scale; BMI: body mass index

The ROC was used to analyse the predictive value of GNRI, NIHSS, BMI, and Albumin to poor outcome. The AUC of those potential predictors was evaluated and plotted (see in Fig. 3 and Table 7). There were all statistically significant differences in the AUC values for GNRI, NIHSS, BMI, and Albumin (all *P* < 0.05). The AUC of GNRI was greater than the AUC values of NIHSS, BMI, or Albumin (0.804 *vs.* 0.738, 0.769, or 0.699) with a statistically significant difference (both *P* < 0.05). In addition, it was found that the AUC of the combined GNRI with NIHSS was largest among all the variables with statistically significant differences (all *P* < 0.05). The AUC of all variables is shown in Fig. 3. The optimal cut-off of the GNRI for predicting poor outcome was 97.69, with a sensitivity of 69.51% and specificity of 77.27%. The AUC of the combined GNRI with NIHSS was 0.855, with a sensitivity of 84.75 and specificity of 72.73%. The details of the ROC parameters of GNRI, Albumin, NIHSS, BMI, and GNRI + NIHSS for poor outcomes are listed in Table 7.

**Table 7:** ROC parameters of GNRI, Albumin, NIHSS, BMI, and GNRI + NIHSS for poor outcome

Variables	Statistical value			Youden	Cut-off	Sensitivity(%)	Specificity(%)
	AUC(95%CI)	***z***	***P***				
**GNRI**	0.804(0.755–0.846)	10.980	< 0.001	0.4678	97.69	69.51	77.27
**NIHSS**	0.738(0.685–0.786)	7.687	< 0.001	0.3248	11	84.75	47.73
**Albumin**	0.769(0.718–0.815)	9.153	< 0.001	0.4029	37.6	61.88	78.41
**BMI**	0.699(0.644–0.749)	5.989	< 0.001	0.3467	22.51	57.40	77.27
**GNRI + NIHSS**	0.855(0.811–0.892)	14.398	< 0.001	0.5748	0.659	84.75	72.73

*Abbreviations*: *GNRI* geriatric nutritional risk index, *NIHSS* National Institute of Health Stroke Scale, *BMI* body mass index

## Discussion

Initially, used for estimating the risk of malnutrition-related complications in an ageing population, the GNRI was the preferred tool for screening the nutritional status of hospitalized elderly individuals [2]. In recent years, the relationship between malnutrition and poor outcome in a variety of diseases has been well developed [22–24]. It is generally accepted that the GNRI has a stronger prognostic effect than traditional nutritional indicators [2, 25].

According to those previous studies, the GNRI was adopted in the present study, and the associations of the GNRI with neurological outcome were explored in populations with stroke. The results showed that lower GNRI or higher nutritional risk grades of GNRI on admission showed an independent correlation with poor neurological function at 1 month since onset of stroke, which was still significant after adjusting for the covariates. In addition, compared with NIHSS, BMI, and Albumin, the predictability of GNRI for poor neurological outcome was more powerful. The GNRI might serve as a promising potential predictor for neurological outcome for patients with stroke at the convalescence stage. To the best of our knowledge, this is the first study regarding the directly predictive property of the GNRI for poor outcomes of convalescence.

With the integration of information derived from both serum albumin and bodyweight, GNRI reflected the nutrition and BMI, and enabled comprehensive assessment of the above variables [24]. As a consequence, the GNRI value was a complementary indicator that improved the diagnostic accuracy and reduced the limitations. The AUC of the GNRI was greater than the AUC of BMI or Albumin (0.804 *vs.* 0.769 or 0.698), with a statistically significant difference (both *P* < 0.05), and increased sensitivity without loss of specificity compared with Albumin or BMI alone.

The reason for the GNRI exhibiting powerful predictive validity in the present study is still unclear. This study merely enrolled patients with stroke in the rehabilitation phase. Albumin not only reflects the state of nutrition, but is also affected by some factors, such as inflammation or disease stress [26]. Therefore, the use of albumin alonely may have a narrow effect for prediction, which was verified by the lower sensitivity of albumin according to our ROC analysis. Nishioka [5] advised using the other NSTs for patients with stroke unless accompanied by assessment of oedema and inflammatory status, as well as excluding the presence of diseases that caused hypoalbuminemia. In this study, a lower proportion of comorbidities, such as pneumonia, other infections, gastrointestinal haemorrhage, and pressure sore, may partly explain the robustness of the GNRI in predicting the neurological outcome, which indicated that further stratification analysis of the predictive efficiency of the GNRI was necessary when accumulating more cases. The relationship between BMI and functional outcome of stroke is complex. In this study BMI had a weak association with the Barthel index. However, a previous study found that functional outcomes after stroke have a nonlinear relationship to patient adiposity [27]. As an effect of obesity paradox, BMI had a U-shaped/J-shaped relationship to unfavorable disability and stroke-related quality of life outcomes [28]. These differing patterns suggest distinct pathophysiologic mechanisms, including greater importance of metabolic reserve against nutritional challenge for survival and greater frequency of atherosclerotic and thromboembolic infarcts in individuals with higher BMI [29]. Limited by small samples, we did not carry out further analysis for BMI.

Studies have confirmed that malnutrition has a negative impact on stroke rehabilitation [3, 30–32]. Several studies have also suggested that enhanced nutritional support is associated with improved independence in activities of daily living and quality of life [33, 34]. However, only a few studies have involved nutritional screening and neurological outcomes. Among them, many previous studies employed length of hospital stay, mortality and complication rates as outcomes of predictive validity [4]; fewer studies used functional outcomes and discharge destination [5]. For inpatient rehabilitation, the latter outcomes may be preferred to the former for predictive validity, similar to using the Barthel index as a functional outcome in this study..

Compared with traditional NSTs such as NRS 2002 [35], the application of the GNRI in stroke patients has the following advantages: (1) the objectivity of the components of the GNRI avoided the difficulty of obtaining subjective indicators when evaluating stroke patients with consciousness or cognitive impairment; (2) the evaluation of the GNRI was very quick and convenient for the needs of stroke patients in bedside and repeated evaluation; and (3) the validity and reliability of the GNRI had been verified in a variety of clinical settings [11, 12, 14, 23, 24]. Thus it was believed that the GNRI might be used as an appropriate NST for identifying nutritional risk and predicting neurological outcome in the rehabilitation setting.

The maximum value of the NIHSS scores among all the participants in this study was 22, and the IQR was 12–16. The scores reflected a moderate-severe severity of stroke, which has the most rehabilitation value [36]. The retrospective review helped us to find that the population with poor outcomes at 1 month after onset presented lower levels of GNRI at baseline compared with the population with good outcomes. According to the grades of GNRI, more cases with poor outcomes distributed in the higher nutritional risk grades of GNRI further verified the positive relationship between GNRI and neurological function (z = 49.268, *P* < 0.001). After adjusting for covariates, Spearman analysis showed a negative relationship between the GNRI and poor outcome. The "r" coefficient in GNRI was higher than it in NIHSS, which indicated a more force weight for GNRI in influence to neurological outcome. This was also verified by line regression analysis with a higher standardization β for the GNRI than for the NIHSS. To exclude the influence of other factors, this study further fitted a multivariate logistic regression analysis, and the negative association between the GNRI and poor outcome of stroke remained (OR = 0.885, *P* < 0.01), which indicated that a high GNRI was a protective factor against poor outcome. When constructing multivariate regression equations, an independent correlation between NIHSS, BMI, and Albumin and the poor outcome of stroke was found (*P* < 0.05), which was the basis of further exploring the predication value of these indexes. To acquire a robust consequence, the relationship between the GNRI grade and poor outcome was further analysed. After adjusting for covariants, the OR of GNRI to poor outcome increased across increasing nutritional risk grades (*P* for trend < 0.001). The OR of the patients with GNRI < 82 was 14 times higher than that of the patients with GNRI > 98, which further verified that malnutrition was a powerful risk factor for poor neurological function. At present, no similar research is available for comparison. Early, a prospective study indicated the OR of a lower GNRI was 2.55 for a poor outcome 3 months after stroke [18]. However the cut-off of GNRI in that study was 92 and the poor outcome was evaluated by modified Rankin Scale (mRS), which was quite different from our study.

The study further explored the predictive effects of GNRI, NIHSS, BMI, and Albumin on poor outcome in convalescence stage for patients with stroke. The ROC analysis found that the AUCs of GNRI and NIHSS were 0.804 and 0.738, respectively, which were good in terms of prediction efficacy. The GNRI had a sensitivity of 69.51%, which was lower than the NIHSS, and had a specificity of 77.27%, which was higher than the NIHSS. NIHSS is generally recognized as a clinical tool for evaluating changes in the condition of patients with stroke. The NIHSS scores was also used as a predicator for neurological function prognosis [17]. However, studies have shown that the prediction efficiency of the NIHSS is insufficient for patients with POCI due to the lack of a relevant index forthe NIHSS [36, 37]. In this study, POCI proportion of stroke lesions was up to nearly 40%, which may affect the predictive efficiency of NIHSS. Our study suggests that the GNRI may remedy the defect of the NIHSS in predicting neurological outcomes for patients with POCI stroke.

To determine the total predictive value of the combined GNRI and NIHSS, we brought GNRI and NIHSS scores simultaneously into bivariate logistic regression fitting and returned logit (p) as a predictive probability, which was used as an independent predictive variable for ROC analysis. It was found that the AUC of the combined GNRI with NIHSS was largest among all the variables with statistically significant differences (all *P* < 0.05). In addition, the combination of the GNRI and the NIHSS scores could improve the sensitivity and specificity compared with the use of either score alone, which indicated that we could acquire more predictive effectiveness by adding the GNRI to regular NIHSS scores when predicting poor neurological outcomes in clinical practice, especially for patients suffering from POCI stroke.

It was difficult to explain the causality of between the GNRI and poor neurological outcome of stroke. There are several plausible mechanisms for this. Patients with stroke often experience a reduction in their body weight, approximately 3 kg, in the acute phase [38]. A decreased body weight could be attributed to muscle atrophy that is primarily caused in paretic limbs and the diminished nutritional intake because of dysphagia [5]. Individuals with a lower GNRI in the study may also include patients who had developed malnutrition before the onset of stroke. Meanwhile, the malnutrition hampers neurological self-recovery and disturbs routine rehabilitation procedures for patients after stroke [4, 5]. Both the factors may be reciprocally causative and even create a vicious cycle. Regardless, nutritional improvement in stroke patients with malnutrition was associated with the resumption of activities of daily living [6].

There are some limitations in this study. First of all, the small sample size of the study affects the statistical efficiency. Second, this study is a cross-sectional study, and the direction of cause and effect between GNRI and poor outcome may be uncertain. As our retrospective study was based on the former data, some potential risk factors and key parameters could not be available, and the dynamic evaluation for GNRI and neurological function was lacking, which affects the persuasiveness of the conclusions. Third, all the serum samples were collected only once from the participants, which may lead to the bias in the analysis.

## Conclusions

In conclusion, for patients with stroke, there was an independent relationship between a lower GNRI on admission and poor outcome at 1 month after the onset of stroke, which suggested that higher nutritional risk grades at baseline may indicate worse neurological function at the convalescence stage. Compared with NIHSS, BMI, and Albumin, GNRI was a competitive indicator for the risk of poor neurological outcome. The predictive property of GNRI for adverse neurological outcomes might be more powerful when combined with NIHSS.

## Data Availability

The datasets used and/or analyzed during the current study available from the corresponding author on reasonable request.

## Abbreviations

NST: Nutrition screening tool
BMI: Body mass index
TACI: Total anterior circulation infarct
PACI: Partial anterior circulation infarction
POCI: Posterior circulation infarction
NIHSS: National Institute of Health Stroke Scale
GNRI: Geriatric nutritional risk index
mRS: Modified Rankin Scale
